# A Case of Extensive Lichen Planus Treated With Deucravacitinib

**DOI:** 10.7759/cureus.71951

**Published:** 2024-10-20

**Authors:** Gretchen D Ball, Alexandra Golant

**Affiliations:** 1 Dermatology, Icahn School of Medicine at Mount Sinai, New York, USA

**Keywords:** classic lichen planus, deucravacitinib, inflammatory skin disease, janus kinase inhibitor, selective tyk2 inhibitor

## Abstract

Lichen planus (LP) is a persistent inflammatory condition that affects the skin and mucous membranes, often significantly impacting quality of life. Recent research has highlighted the potential of Janus kinase (JAK) inhibitors as an effective treatment for LP. We report a case of a 52-year-old woman with a history of hypertension, obesity, and hypothyroidism who presented with a widespread, itchy, and scaly rash that was present for one month and affected 80% of her body surface area (BSA). A biopsy from the right lower extremity confirmed LP. After two months of treatment with deucravacitinib, the patient demonstrated significant improvement, with BSA involvement reduced to 20% and erythema notably diminished. To our knowledge, this is the first documented case of extensive cutaneous LP successfully managed with deucravacitinib. Unlike other JAK inhibitors, deucravacitinib selectively targets tyrosine kinase 2 (Tyk2), offering focused modulation of the immune response while minimizing broader adverse effects. This specificity provides deucravacitinib with a more favorable safety profile, making it a potentially ideal option for the long-term management of chronic dermatologic conditions like LP, which often require extended treatment.

## Introduction

Lichen planus (LP) is a chronic inflammatory disorder of the skin or mucous membranes that significantly impacts patient quality of life. Cutaneous LP is characterized by pruritic, thin, and violaceous papules and plaques that often cause considerable discomfort. LP can also manifest in several morphological and anatomical variants, including oral, nail, and genital LP. The root cause of LP remains largely unknown, but it is widely recognized as a T-cell-mediated autoimmune disorder, with potential triggers including viral infections like hepatitis C, contact allergens, medications, genetic susceptibilities, or other environmental stimuli [1].

Several underlying immune pathways have been identified in the pathogenesis of LP. The condition is characterized by a pronounced infiltration of CD8+ cytotoxic T-cells in the dermis and epidermis, which are believed to target basal keratinocytes presenting altered exogenous or self-antigens. T-cell activation triggers the release of a broad array of cytokines, initiating inflammatory cascades that foster the proliferation of T helper type 1 (Th1) and T helper type 17 (Th17) cell populations. These are mediated by interferon-gamma (IFN-γ), tumor necrosis factor-alpha (TNF-α), and interleukins (IL)-6, -12, -17, -21, and -23, among other immune factors. The resulting cellular processes lead to the apoptosis and necrosis of keratinocytes, producing the classic clinical features of LP [2].

Topical steroids are the first-line treatment for localized cutaneous LP, whereas systemic and intralesional steroids are reserved for more extensive or resistant cases. Additional treatment options include oral retinoids, phototherapy, and systemic immunosuppressants like cyclosporine, methotrexate, or azathioprine [3]. Although these treatments can alleviate the significant pruritus and inflammation characteristic of LP, targeted therapy for the disorder remains elusive.

Recent studies have highlighted the potential of Janus kinase (JAK) inhibition as an effective treatment for LP, particularly given the involvement of both Th1 and Th17 pathways in the disease [4]. Tyrosine kinase 2 (Tyk2) is a protein in the JAK family involved in the signaling of interferons, IL-12, and IL-23, all of which are implicated in the pathogenesis of LP [5]. Deucravacitinib, a Tyk2 inhibitor, has been FDA-approved for adults with moderate to severe plaque psoriasis and is currently under investigation for efficacy in other immune-mediated diseases. This report aims to highlight the clinical efficacy of deucravacitinib in treating a case of extensive cutaneous LP and to discuss its potential as a novel treatment option.

## Case presentation

A 52-year-old female with a past medical history of hypertension, obesity, and hypothyroidism presented to the dermatology clinic for evaluation of an extensive, pruritic, and scaly rash that had been present for one month. A recent prior biopsy of the rash on the right lower extremity confirmed a diagnosis of LP. Physical examination revealed diffuse violaceous scaly plaques over her trunk and extremities, covering roughly 80% of her body surface area (BSA). No nail or oral involvement was detected.

Prior attempts to manage the condition with triamcinolone 0.1% cream and hydroxyzine for approximately three weeks had not alleviated her symptoms. A comprehensive assessment provided no evidence of known triggers of LP, including negative hepatitis C virus serologies. After discussing therapeutic options and considering the patient’s goal to avoid systemic steroids or immunosuppression, the decision was made to initiate treatment with deucravacitinib at a dose of 6 mg once daily, in addition to topical ruxolitinib 1.5% cream twice daily.

**Figure 1:**
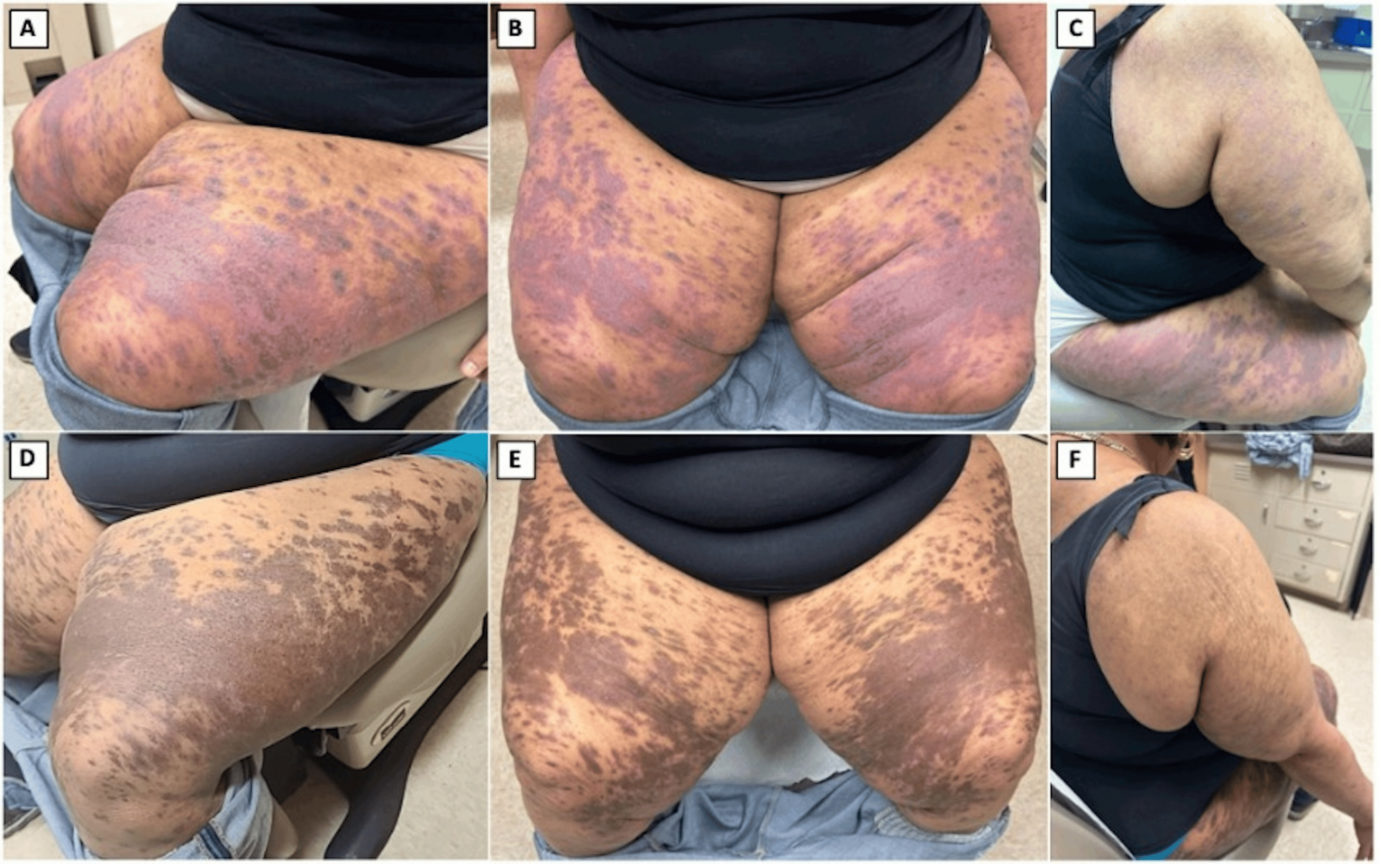
Extensive lichen planus before (A-C) and after (D-F) two months of treatment with deucravacitinib

Upon reevaluation five weeks later, the patient exhibited improvement with a reduction in estimated BSA involvement to 50%. We opted to continue deucravacitinib 6 mg daily and add triamcinolone 0.1% ointment. Two months after starting deucravacitinib, the patient demonstrated substantial improvement, with a reduction of BSA involvement to 20% and a notable reduction in erythema, with mostly hyperpigmented patches remaining (Figure 1). The patient also reported a significant decrease in itch severity, indicating a favorable response to deucravacitinib.

## Discussion

Treatment of cutaneous LP with deucravacitinib has rarely been reported. In 2022, Motamed-Sanaye et al. identified 56 patients across 15 studies treated with JAK inhibitors-specifically tofacitinib, upadacitinib, baricitinib, and topical ruxolitinib-for LP and its clinical variants [6]. These treatments resulted in the partial or complete resolution of LP lesions in 73.3% of these cases, underscoring the potential of JAK inhibition in LP management.

Deucravacitinib, unlike non-selective JAK inhibitors such as tofacitinib, targets the immune response through selective Tyk2 inhibition. This specificity minimizes its impact on hematopoietic and broader immune processes, granting deucravacitinib a more advantageous safety profile than other JAK inhibitors. For this reason, deucravacitinib may be particularly well suited for sustained treatment of chronic dermatologic conditions like LP, which often require extended therapy. In our case, deucravacitinib was well tolerated by the patient with no notable adverse events.

Thus far, four cases of isolated oral LP successfully treated with deucravacitinib have been reported. One case involved a 52-year-old woman with erosive oral LP who started 6 mg of deucravacitinib daily and reported significant symptom relief in the first five days of treatment, remaining symptom-free at her six-month follow-up appointment [7]. In a separate case series, three patients with chronic erosive oral LP were treated with deucravacitinib 6 mg daily for 12 weeks, and all experienced an improvement in mucosal lesions without significant symptoms or serious adverse events. Immunological assessments indicated that the results were consistent with the known effects of Tyk2 inhibition; specifically, there was a decrease in the production of IFN-γ, IL-2, and IL-17, alongside an increase in IL-4 production [8]. While the immunopathogeneses of oral and cutaneous LP are not identical, both conditions share overlapping inflammatory pathways. The consistent clinical improvements observed in the four cases of oral LP and our report suggest that deucravacitinib may effectively treat various manifestations of the disease.

## Conclusions

In conclusion, this case highlights the potential efficacy of deucravacitinib in the management of cutaneous LP. Its selective Tyk2 inhibition offers a targeted therapeutic approach with a favorable safety profile, making it particularly advantageous for chronic dermatologic conditions like LP. The absence of notable adverse events in this case mirrors findings from previous reports on its use in oral LP. Given the positive clinical outcomes and strong safety profile observed, deucravacitinib could represent a significant advancement in the treatment of LP. However, further large-scale studies are warranted to confirm its long-term efficacy and safety across a broader LP patient population.

## References

[1] Arnold DL, Krishnamurthy K (2024). Lichen planus. StatPearls [Internet].

[2] Vičić M, Hlača N, Kaštelan M, Brajac I, Sotošek V, Prpić Massari L (2023). Comprehensive insight into lichen planus immunopathogenesis. Int J Mol Sci.

[3] Boch K, Langan EA, Kridin K, Zillikens D, Ludwig RJ, Bieber K (2021). Lichen planus. Front Med (Lausanne).

[4] Pietschke K, Holstein J, Meier K (2021). The inflammation in cutaneous lichen planus is dominated by IFN-ϒ and IL-21-A basis for therapeutic JAK1 inhibition. Exp Dermatol.

[5] Muromoto R, Oritani K, Matsuda T (2022). Current understanding of the role of tyrosine kinase 2 signaling in immune responses. World J Biol Chem.

[6] Motamed-Sanaye A, Khazaee YF, Shokrgozar M, Alishahi M, Ahramiyanpour N, Amani M (2022). JAK inhibitors in lichen planus: a review of pathogenesis and treatments. J Dermatolog Treat.

[7] Vu M, Abdin R, Issa NT (2023). Treatment of oral lichen planus using deucravacitinib. JAAD Case Rep.

[8] Stolte KN, Mesas-Fernández A, Meier K, Klein EK, Dommisch H, Ghoreschi K, Solimani F (2024). TYK2 inhibition with deucravacitinib ameliorates erosive oral lichen planus. Exp Dermatol.

